# Urgent Hospital Admissions Caused by Adverse Drug Reactions and Medication Errors—A Population-Based Study in Spain

**DOI:** 10.3389/fphar.2020.00734

**Published:** 2020-05-21

**Authors:** Gina Mejía, Miriam Saiz-Rodríguez, Beatriz Gómez de Olea, Dolores Ochoa, Francisco Abad-Santos

**Affiliations:** ^1^Clinical Pharmacology Department, Hospital Universitario de La Princesa, Instituto Teófilo Hernando, Facultad de Medicina, Universidad Autónoma de Madrid (UAM), Instituto de Investigación Sanitaria La Princesa (IP), Madrid, Spain; ^2^UICEC Hospital Universitario de La Princesa, Plataforma SCReN (Spanish Clinical Research Network), Instituto de Investigación Sanitaria La Princesa (IP), Madrid, Spain; ^3^Research Unit, Fundación Burgos por la Investigación de la Salud, Hospital Universitario de Burgos, Burgos, Spain

**Keywords:** adverse drug reaction, medication error, urgent admission, pharmacovigilance, emergency department

## Abstract

**Background:**

Adverse drug reactions (ADR) are a public health issue, due to their great impact on morbidity, mortality, and economic cost.

**Objective:**

We aimed to study the percentage of patients admitted urgently as a result of an ADR, considered serious adverse event, or medication error. Also, we intended to identify possible risk factors which would lead to improvements in the prescription and use of medications.

**Methods:**

This is a retrospective observational study conducted during February 2019, including patients admitted through the emergency department in our hospital. We evaluated the medical records of those with suspected ADR diagnoses to perform a descriptive analysis of the demographic characteristics. Moreover, after applying the Spanish Pharmacovigilance System causality algorithm, we performed a descriptive analysis of the identified ADR and the drugs involved. We also investigated those cases suspected of being a medication error.

**Results:**

During the study period, 847 patients were urgently hospitalized. From those, 71 (29 women and 42 men) were admitted due to an ADR (8.4%, 95% CI 6.5%–10.3%). The mean age was 73 ± 15.9 years old and the mean number of prescribed medications was 7.3 ± 3.6 drugs/patient on admission. The most frequent ADR were opportunistic infections due to antineoplastic and immunomodulator drugs, and bleeding due to antiaggregants and anticoagulants. Five suspected medication errors occurred, being the incidence 0.6% (95% CI 0.08%–1.12%) of total admissions.

**Conclusions:**

8.4% of urgent admissions were attributed to an ADR. Age (75% of patients were ≥ 65 years old), comorbidities and polymedication were the main risk factors. Although medication errors had a very low incidence (0.6% of urgent admissions), they were preventable and should be considered as a focus for action.

## Introduction

Drugs are capable of preventing, relieving, or improving human health. Mostly, they are substantially beneficial and have meant the greatest advance for society, improving human quality of life, and life expectancy. However, drugs are not exempt from risks. During clinical development, prior to marketing authorization, the number of patients exposed to investigational drugs is very low. Therefore, rare adverse reactions are not correctly identified. For this reason, the pharmacovigilance strategy of adverse events monitoring in daily clinical practice is a cornerstone for prompt adverse drug reaction (ADR) identification and prevention.

An ADR is any unintended, harmful response to a drug. It includes not only unplanned harmful effects resulting from the authorized use of a drug at normal doses, but also related to medication errors and uses outside the terms of the marketing authorization, including misuse, overdose and drug abuse (Spanish Ministry of Health). ADR are a public health issue, due to their great impact on morbidity, mortality and economic cost (Patel et al., 2007). Several meta-analyses have studied the frequency of ADR as a cause of hospitalization in Western countries, raising a range of 3.7%–5.3% of hospital admissions caused by an ADR (Pedrós et al., 2014). However, there is still a considerable variability between studies since some report a rate of 4.2% (Pedrós et al., 2014), 4.8% (Garijo et al., 1991), 6.7% (Lazarou et al., 1998), or even 8.8% (Ahern et al., 2014) of ADR-caused hospital admissions. This inconsistency might be due to different study design, the type of events studied, the definition of ADR used, the methods of case identification, variations in the causality algorithms, the study duration, etc.

In the USA, ADR are diagnosed in approximately 4.7% of hospitalized patients. However, according to Crispo et al., many cases are readmissions, patients who were discharged and who are readmitted within 45 days as a result of an ADR (Crispo et al., 2019). Indeed, the risk of suffering an ADR after discharge is five times greater than the risk during hospitalization (Crispo et al., 2019).

Some ADR may be due to medication errors. Namely, incidents that were preventable and occur at any stage of the use process, that can produce harm to patients. These include errors in: prescribing, communication, labelling, packaging, naming, preparation, dispensing, distribution, administration, education, and monitoring. These errors often occur at several levels at once, so they should be considered as system errors, and never as human errors. It is estimated that for each hospitalized patient there are 0.9 medication errors per day (Pastó-Cardona et al., 2009). Certainly, between 44,000 and 98,000 deaths per year were attributable to medical errors or failures in the health system (Pastó-Cardona et al., 2009). However, a more recent study estimated that 1.1% of deaths are due to medical errors which, extrapolated to all hospital admissions recorded in the USA, reached 400,000 deaths per year, four times more than the previously valued (Makary and Daniel, 2016). Currently, medical errors are still one of the most important causes of death in the USA, ranking third after cardiovascular diseases and cancer (Makary and Daniel, 2016; Crispo et al., 2019).

Medication consumption is increasing in recent years, consequently, ADR and medication errors might also be on the rise. A high number of admissions can be attributed to ADR, in which the patient’s health might be at risk or even cause life-threatening situations. The impact of these ADR on public health is noticeable on several levels: quality of health care, patient’s quality of life, and economic expenditure.

For these reasons, we aimed to study the percentage of patients admitted urgently as a result of a serious ADR, and how many are due to medication errors. We aimed to identify possible patterns and risk factors which would lead to improvements in drug prescription, administration, consumption, and monitoring. Therefore, the amount and seriousness of such ADR might be reduced.

## Materials and Methods

### Study Population and Study Design

A retrospective observational study was conducted at the Clinical Pharmacology Department of Hospital Universitario de La Princesa, a third-level university hospital in Madrid (Spain). It has approximately 564 beds and covers a population of 323,000 individuals. It offers all medical and surgical specialties, except for pediatrics and obstetrics-gynaecology. The study period was 28 days and included all patients who were admitted trough the emergency room during February 2019. Patients with scheduled admissions, emergency observation stays, admissions of less than 24 h, and transfers from other centres were excluded.

### Sample Size Calculation

For a 95% confidence level (CI), and an accuracy of +/- 2 percentage units, the number of medical records required for the study was 507, with a population percentage predicted to be around 5%. The percentage of non-assessable patients was calculated to be 10%.

Given that the Emergency Department of Hospital Universitario de La Princesa admits about 8,000 patients a year, the information was collected over a period of 28 days (approximately 600 admissions). From those, the medical records of patients with suspected ADR were thoroughly reviewed for causality assessment and identification of medication errors.

#### Suspected ADR Diagnostics

All admissions with a suspected ADR-related diagnosis, according to a previously pre-defined list (see Pedrós et al., 2014), were reviewed to assess whether there was any suspected drug responsible for the admission. The pre-defined list includes all diseases or syndromes potentially caused by drugs. Demographic variables were collected from patients with suspected ADR: age, sex, weight, and height, and diagnosis at admission. In addition, special attention was paid to the usual treatment of each patient, analysing each drug to detect the potential responsibility for the ADR.

In every patient with a suspected ADR, the causality algorithm published by the Spanish Pharmacovigilance System (SEFV) (Aguirre and García, 2016), which is a modification of the one published by Karch and Lasagna (Karch and Lasagna, 1977), was applied to each suspected drug by two investigators (GM and BG) and when the score was different an agreement was sought.

The SEFV algorithm comprises seven criteria, which are assessed trough every drug-adverse effect pair: (1) Time sequence. Chronology between the start of treatment with the suspected drug(s) and the appearance of the adverse effects. The compatibility of this sequence with the mechanism of action of the drug and/or with the physio-pathological process of the adverse effect is analyzed; (2) Previous knowledge of the adverse effect in the literature; (3) Effect of withdrawal: evolution of the adverse effect after withdrawal of the suspected medication; (4) Re-exposure: effect of re-administration of the suspected medicinal product; (5) Alternative causes: existence of an alternative cause, a non-pharmacological explanation for the observed effects; (6) Contributing factors favouring the causal relationship (e.g., renal failure and relative overdose of a drug with predominantly renal elimination); (7) Complementary exploration: drug levels in a biological fluid, biopsy, positive radiological examinations, positive specific skin tests, etc.

Based on the obtained scored, the causal relationship was classified as: unrelated (< 1), conditional (1–3), possible (4–5), probable (6–7), and defined (> 7) (Aguirre and García, 2016). Only those adverse events classified as possible, probable, or defined were considered drug-related. Therefore, possible, probable, or defined adverse events were merged into the category ADR, so they were grouped together for the analysis.

### Ethics

The project obtained its approval from the Ethics Committee for Research on Medicines (CEIm) of Hospital Universitario de La Princesa. As all the information was registered from the electronic medical record without interviewing the patients, it was not necessary to ask for the patients’ informed consent.

The researchers respected the confidentiality of every data obtained during the conduct of the study. No data were collected that could identify the patients.

#### Data Collection and Information Sources

The information was registered only from the hospital’s computer system database, by reviewing patients’ medical records. There was no need to interview patients to obtain additional information; therefore, informed consent was not required. Data collection was performed without causing any alteration in the information system or any variation in usual clinical practice.

### Data Analysis

The statistical analysis was accomplished using the SPSS 22.0 statistical software (SPSS Inc., Chicago, Illinois) and Microsoft Excel 2010. The main variable was the incidence of ADR-caused hospital admission. The percentage of ADR due to medication errors was also calculated. The characteristics of patients admitted by an ADR and the main drugs involved were described.

## Results

### Suspected ADR and Patients’ Characteristics

During the whole study period, 847 patients were admitted urgently to Hospital Universitario de La Princesa. These patients’ diagnoses were reviewed according to the list published by Pedrós et al. (2014). In all, we found 232 patients whose admission could be due to an ADR. Then, the exhaustive review of these 232 patients’ medical records yielded the necessary information to establish the ADR-related diagnosis.

After this medical records revision, the number of patients who could have possible ADR was reduced to 95. The remaining patients were excluded due to incompatibility with the diagnosis of an ADR. The criteria for exclusion were: not receiving routine treatment or no medication suspected, the time sequence was found to be within a post-operative range and in relation to the surgery they had undergone, or the condition on admission was triggered by a community-acquired infection, with the exception of opportunistic infections.

The diagnoses of suspected ADR are shown in **Table 1**. The most frequent were haemorrhages or hematomas (20%), opportunistic infections (16.8%), and heart failure decompensation (16.8%).

**Table 1:** Suspected adverse drug reaction (ADR) diagnoses in our study population of urgent admissions (n = 847).

Diagnosis	N	Percentage
Haemorrhages/hematomas	19	20.0
Opportunistic infections	16	16.8
Heart failure decompensation	16	16.8
Vomiting or diarrhoea	9	9.5
Low level of consciousness or bronchial aspiration	8	8.4
Arrhythmias or heart block	7	7.4
Renal failure	6	6.3
Autolytic attempt	3	3.2
Pulmonary thromboembolism	2	2.1
Others	9	9.5
Total	95	100

Others category include individual cases of: respiratory failure, intoxication, hypotension, exanthema, hyponatremia, dehydration, pancreatitis, liver toxicity, and syncope.

From these 95 patients, 46 were women and 49 were men, with a mean age of 73 ± 15.9 years old, median age 75 (range 26–100) years old, 75% of patients were older adults (> 65 years old), with a similar distribution between both sexes. Regarding cardiovascular risk factors, 70.5% presented one or more, being high blood pressure (58.9%), dyslipemia (36.8%), and diabetes mellitus (16.8%) the more frequent.

About the unhealthy lifestyle habits: 30 had smoked and 13 were still smoking, accumulating an average pack-years index of 45.2 and a standard deviation of 28.9 (ranging from 2 to 100). Regarding the history of alcohol abuse, we found 13 patients who showed an important consumption, 6 of which no longer maintained it in the present. Only 15 patients admitted for suspected ADR did not present any cardiovascular risk factor or unhealthy lifestyle. It is worth noting that this group of patients received an average of 7.3 drugs per person, with a range of 1 to 17.

A total of 155 drugs that could be responsible for the adverse reactions in the study were collected from the 95 patients. We found that 46 patients had only one suspected drug, 39 patients had 2 suspected drugs and 10 patients had 3 or more.

The Spanish Pharmacovigilance System causality algorithm (Aguirre and García, 2016) was applied to each of the suspected drugs. As shown in **Tables 2** and **3**, if we consider an ADR those events with a possible, probable or definite causal relationship (score ≥ 4) for at least one drug, we found that 71 patients (74.7%) had been admitted as a result of an ADR. Besides, more than one drug could have been involved in the reaction since the causality algorithm revealed 47 possible, 47 probable, and 1 definite causal relationships. **Table 3** depicts the main drugs categories found in our study population, being the most frequent antihypertensives and diuretics (18.7%) and antineoplastics (16.8).

**Table 2:** Evaluation of causality of the drugs received by the patients with suspected adverse drug reaction (ADR) (n = 95).

	Number of drugs	Percentage
**Unrelated**	9	5.8
**Conditional**	51	32.9
**Possible**	47	30.3
**Probable**	47	30.3
**Defined**	1	0.6
**Total**	155	100

**Table 3:** Drugs potentially involved in the adverse drug reaction (ADR) and their categorization in each causal group.

Type of drug	Causality					Total
	Unrelated	Conditional	Possible	Probable	Defined	
**Antihypertensives and diuretics**	3	16	5	5	0	**29**
**Antineoplastics**	0	7	7	12	0	**26**
**Immunomodulators**	2	3	2	5	0	**12**
**Antiplatelet agents**	0	0	2	8	1	**11**
**Anti-epileptics**	2	3	5	0	0	**10**
**Atypical antipsychotics**	0	3	5	2	0	**10**
**Anticoagulants**	0	0	6	1	0	**7**
**Beta-blockers**	0	2	4	0	0	**6**
**NSAIDs**	0	2	3	1	0	**6**
**Antibiotics**	0	2	0	4	0	**6**
**Antidepressants**	0	4	0	0	0	**4**
**Antiretrovirals**	0	1	0	3	0	**4**
**Benzodiazepines**	1	2	0	0	0	**3**
**Opioids**	0	0	1	1	0	**2**
**Others**	1	6	7	5	0	**19**
**Total**	**9**	**51**	**47**	**47**	**1**	**155**

NSAIDs, nonsteroidal anti-inflammatory drugs. The category “others” include: zolpidem, pentoxifylline, propofol, digoxin, desogestrel/etinylestradiol, granisetron, hydroxychloroquine, memantine, rivastigmine, galantamine, lithium, haloperidol, fenofibrate, levothyroxine, tamsulosin-dutasteride, and the inhaler formoterol-beclomethasone.

Therefore, in the population of our sample, the percentage of urgent admissions as a result of an ADR is 8.4% with a 95% CI of 6.5%–10.3%.

From the 71 patients admitted for an ADR, 29 were women and 42 were men, with an average age of 73 years old and a mean intake of 7.3 drugs per patient. These results were similar to those from the total of 95 patients with suspected ADR. However, the incidence of cardiovascular risk factors and unhealthy lifestyle habits were slightly higher in this group of patients with ADR: 61% had high-blood pressure, 38% dyslipemia, and 20% diabetes mellitus. Moreover, 13% smoked and 37% had smoked in the past (accumulating an average pack-years index of 48 (ranging from 2 to 100), 13% had an abuse alcohol history. Finally, only nine patients lack any cardiovascular risk factor or unhealthy lifestyle.

**Table 4** shows the incidence of ADR in each group of drugs. The majority of ADR were found in the antineoplastics group (20%), followed by the category “others” (12.6%), antiplatelet agents (11.6%), and antihypertensives and diuretics (10.5%). None of the ADR was due to an allergy.

**Table 4:** Drugs suspected to cause urgent hospital admission by adverse drug reaction (ADR) in our study population.

Type of drug	Number of patients	Percentage	Description of ADR
**Antineoplastics**	19	20.0	Opportunistic infections: 18; vomiting or diarrhea: 1.
**Antiplatelet agents**	11	11.6	Haemorrhages/hematomas: 11.
**Antihypertensives and diuretics**	10	10.5	Heart failure decompensation: 5; renal failure: 4; arrhythmias or heart block: 1
**Anticoagulants**	7	7.4	Haemorrhages/hematomas: 6; pulmonary thromboembolism: 1.
**Immunomodulators**	7	7.4	Opportunistic infections: 7.
**Atypical antipsychotics**	7	7.4	Low level of consciousness or bronchial aspiration: 6; liver toxicity: 1.
**Anti-epileptics**	5	5.3	Low level of consciousness or bronchial aspiration: 4; vomiting or diarrhea: 1.
**Beta-blockers**	4	4.2	Heart failure decompensation 3; arrhythmias or heart block: 1.
**NSAIDs**	4	4.2	Heart failure decompensation: 1; renal failure: 2; hyponatremia:1.
**Antibiotics**	4	4.2	Haemorrhages (haemorrhagic colitis): 1, vomiting or diarrhea: 2; exanthema: 1.
**Antiretrovirals**	3	3.2	Vomiting or diarrhea: 2; pancreatitis 1.
**Opioids**	2	2.1	Low level of consciousness or bronchial aspiration: 1; vomiting or diarrhea: 1.
Others*	12	12.6	Heart failure decompensation: 3; low level of consciousness or bronchial aspiration: 4; pulmonary thromboembolism: 1; vomiting or diarrhea:1; arrhythmias or heart block: 1; hypotension: 1; pancreatitis 1.
Total	95	100	

NSAIDs, nonsteroidal anti-inflammatory drugs. *Drugs: tamsulosin-dutasteride (Heart failure decompensation), zolpidem, memantine, rivastigmine and galantamine (low level of consciousness or bronchial aspiration), contraceptive (pulmonary thromboembolism), granisetron (diarrhoea), digoxin (arrhythmia), propofol (hypotension), and fenofibrate (pancreatitis).

### Medication Errors

We found five potential medication errors (**Table 5**), three of which belonged to the group of 71 patients who had been admitted for an ADR, i.e., 4.2%. And of the total patients studied in February, they represented 0.6% (95% CI 0.08%–1.12%).

**Table 5:** Medication errors found in our study population of patients urgently admitted to the hospital (n = 847).

Drug	ADR	Error
Trimethoprim/sulfamethoxazole	Opportunistic infection	Withdrawal
Acenocumarol	Pulmonary thromboembolism	Withdrawal
Anticoagulant	Deep vein thrombosis	Absence of prescription
Insuline	Ketoacidotic coma	Misuse
Valproic acid	Intoxication	Duplicate prescription

ADR, adverse drug reaction.

In two patients, the event occurred when the drug was withdrawn (Septrin^®^ and Sintrom^®^). Another patient had a grade 2 prothrombotic syndrome and possibly needed anticoagulation. Another patient presented valproic acid poisoning due to a duplication of the prescription (a prescription of 600 mg every 12 h and another of 1,000 mg every 12 h) and the last one was diabetic and did not inject the prescribed insulin due to ignorance of the possible consequences.

## Discussion

### Adverse Drug Reactions

A serious ADR is any adverse reaction that results in death, may be life-threatening, requires hospitalization of the patient or prolongation of existing hospitalization, causes significant or persistent disability or invalidity, or constitutes a congenital anomaly or birth defect (Spanish Ministry of Health, 2013). So, all ADR considered in this study are serious adverse reactions.

Our research revealed an ADR-related urgent admission rate of 8.4% (95% CI: 6.5%–10.3). Although our results resembled of those from Ahern et al, who found a rate of 8.8% of urgent admissions attributable to an ADR in Irish population (Ahern et al., 2014), our results are notably higher than the previously reported for Spanish population (4.2%–4.8%) (Garijo et al., 1991; Pedrós et al., 2014). However, the overall incidence of serious ADR (causing hospitalization) in the US was reported to be 6.7% (95% CI 5.2%–8.2%) in the meta-analysis performed by Lazarou et al. already in 1998 (Lazarou et al., 1998). Other previous studies evaluated similar settings, but with different study design, such as the one by Taylor et al. (2020), which is a prospective study that evaluates problems related to medication before, during and after the patient goes to the emergency department. This study does not analyze urgent hospital admissions caused by ADRs, which is the main objective of our study. Moreover, a recent study by Just et al. (2020), although describes ADRs leading to emergency department visits and the drugs related with them, it provides different data from ours since they did not calculate the rate of ADR-caused hospital admissions.

Recently, Schurig et al. calculated the percentage of suspected ADR cases among all patients presenting to the emergency room during a 30-day period of observation, accounting for 6.5% of the total visits (Schurig et al., 2018). However, this study does not provide data about hospital admissions. Although similar to ours, it does not show how many of the admitted patients are due to ADRs and it does not evaluate medication errors.

The study by Hamed et al. only collected 159 cases of ADRs from 113,272 emergency department visits (Hamed et al., 2017), accounting for the 0.14%, which might be insufficient. In our study, 11% of the urgent hospital admissions were categorized as possible ADR-caused that reduced to 8.4% after applying the causality algorithm. Moreover, this study does not provide data on the incidence of ADR-caused hospital admissions.

On the other hand, some recent studies evaluate the incidence and risk factors of ADR attended in the emergency department that are related to a specific drug, such as Tanreqing injection, a traditional Chinese medicine commonly used in China (Li et al., 2020). This study is not comparable to ours, which provides much more general data that can be more easily extrapolated to the general population.

Finally, the relevant study by Budnitz et al. described emergency hospitalizations for recognized ADR in older adults (Budnitz et al., 2011). This study differs from ours mainly in the study design, since it was carried out from an electronic registry without systematically reviewing all the patients admitted as we did. For this reason, our data is more reliable, especially if we want to calculate the incidence of admissions caused by ADRs. Moreover, it was conducted 9 years ago, so it is necessary to continue providing data.”

The fact that our rate is higher in our population compared to the reference Spanish study, could be explained by some differences in the study population included. Firstly, our population is less heterogeneous than the general, since our hospital does not attend children but attends a majority of aged population, compared to other hospitals from the same region of Madrid. In our study, the mean age was 73.0 ± 15.9, ranging from 26 to 100 years old. As previously known, age is itself a risk factor for developing ADR due to pharmacokinetic and pharmacodynamic changes, and the pace of population ageing is increasing. These facts explain the high prevalence of comorbidities and polymedication found in our study (7.3 drugs/person, with a range of 1–17), also considered risk factors themselves (Leendertse et al., 2008). However, the study by Pedrós et al. included a similar population with a mean age of 75 years old (Pedrós et al., 2014). Indeed, our results confirm those from Kojima et al., who reported that in geriatric patients the incidence of ADR is significantly associated with polypharmacy (especially ≥7 drugs) and emergency admission (Kojima et al., 2019).

There were a few cases difficult to assess, as the relation between immunosuppressants (steroids) and the appearance of opportunistic infections, since in many cases these ADR were not displayed in the drug’s label. To avoid possible bias, we included those cases in the study and applied the causality algorithm. Moreover, the same occurred when analyzing the hospital admissions due to reactions such as decompensation of heart failure, low level of consciousness, arrhythmias, or renal failure. Those cases were controversial, since these reactions are very frequent alterations in older adults and can be explained by the natural evolution of their basal pathology. Again, to avoid possible bias, it was decided to give the same value to both variables when applying the causality algorithm. In admissions caused by a haemorrhage, those triggered by a hypertensive crisis or trauma were excluded, but those that arise spontaneously during antiaggregation or anticoagulant therapy were analysed, all finally categorized as ADR. Finally, autolytic attempts are included within the definition of ADR in two situations: (i) cases in which a drug may promote suicidal ideation and (ii) those cases in which the autolytic attempt is carried out with a drug overdose. For this reason, benzodiazepine intoxication was added to the list of established ADR.

This higher degree of case unravelling and recognition, along with a wider definition of an ADR, could also have led to a higher ADR-related admission rate (8.5% vs. 4.2%–4.8% from previous studies) (Garijo et al., 1991; Pedrós et al., 2014). Further approaches are needed to shed light on this controversial rate.

Regarding sex, the study by Ruiter et al. specify that being a female is a risk factor for the development of an ADR (Ruiter et al., 2012). However, in both our study and the one performed by Pedrós et al., no significant differences between sexes were found (Pedrós et al., 2014). In our study, from the 71 patients admitted for an ADR-related condition, 42 were men. However, we are not able to determine that being a male is a risk factor for ADR development.

The most frequently reported reactions were opportunistic infections, caused by antineoplastic and immunomodulatory treatments, and haemorrhages or hematomas, as a consequence of antiaggregation and anticoagulant therapy. Unlike the study by Pedrós et al., which was carried out in a hospital where there was no oncology service (Pedrós et al., 2014). This could explain the absence of opportunistic infections in their study, in favour of a higher incidence of renal and urinary disorders followed by bleeding of digestive origin (Pedrós et al., 2014). In the study by Zhang et al., anticoagulants were the most common medications contributing to ADR-related hospitalisation, followed by opioid analgesics (Zhang et al., 2019). In concordance to our results, the study by Just et al. reported that antineoplastic/immunomodulating agents had the highest odds ratio for being suspected for an ADR, followed by antithrombotics (Just et al., 2020). Schurig et al. also reported antithrombotic and antihypertensive agents as the most commonly suspected cause of ADR (Schurig et al., 2018).

Furthermore, it should be noted the high number of ADR possibly caused by antihypertensive and diuretic drugs that may be linked, for example, to a change in the patient’s prescriptions by the primary care physician, with potential adverse interactions with existing medications. In the study by Ognibene et al., diuretics were the most common ADR causing drugs, followed by antithrombotics and central nervous system-active drugs (Ognibene et al., 2018). However, in our study, after applying the causality algorithm, we observed that they were not the real cause of the adverse reactions *a priori* attributed to them, as described in other study (Ahern et al., 2014). Moreover, suspected ADR from antidepressants or benzodiazepines have been discarded. While, in the case of antibiotics, antiretrovirals, and beta-blockers, they have proved to be responsible for almost all reactions that were attributed to them.

All the drugs included have been marketed for a long time and are considered safe. However, any drug is free from causing an ADR, especially when prescribed to those patients more susceptible of developing an adverse reaction, such as older adults. In addition, this type of patients usually receives several concomitant treatments, which might interact between them. Therefore, measures should be established for stricter control over these patients, by monitoring their pathologies and reducing the number of prescribed drugs to the minimum possible. Almost 45% of the ADR have been reported to be preventable (Al Damen and Basheti, 2019). Certainly, insufficient monitoring was the cause of almost 30% of those (Al Damen and Basheti, 2019).

It is of great importance the role of primary care physicians (PCPs) in the prevention of ADR. Most of PCP are aware of the importance of a close attention when prescribing several drugs to a patient (Raguz Lucic et al., 2018). However, it must be taken into account the difficultness of properly supervise the use of multiple medications due to the potential ADR and drug-drug interactions (Ko et al., 2008). A recent study by Raguz Lucic et al. found that 92.8% of surveyed PCPs explained the possible ADR and drug-drug interactions to special groups of patients (i.e., pregnant women or elderly patients) (Raguz Lucic et al., 2018). However, from 10 questions regarding their knowledge about drug-drug interactions and ADR, the median number of correct response was 5 (range 4–7) (Raguz Lucic et al., 2018). Although PCP are aware of the importance of counseling practices about ADR and interactions (Raguz Lucic et al., 2018), further formation is needed. Furthermore, also pharmacists could be essential for monitoring the potential ADR in general practice, being the collaboration between PCP and pharmacists of mutual benefit (Shulman et al., 1981).

Moreover, pharmacovigilance is an area where PCP should be proactive and remind doctors of their moral duty to inform possible ADR. Without a good pharmacovigilance system, it would be impossible to assess long-term adverse effects of drugs and other possible ADR that would not be highlighted in time to avoid a hospital admission or even save lives.

### Medication Errors

In Spain, there are 1.4 medication errors per hospitalized patient, around 23.6 per 100 admissions per year, 6.1 adverse effects, and 5.5 potential adverse effects per 100 admissions, although only 5% cause appreciable damage (Pastó-Cardona et al., 2009). In our study, the incidence of hospital admission due to a medication error reached only 0.6% (95% CI 0.08%–1.12%), much lower than 4.7%–5.3% previously reported (Pastó-Cardona et al., 2009). In our study, we found 1 case of double prescription, much lower than the 10 cases (6.3%) found in a previous study (Hamed et al., 2017).

A recent study in 6,427 ADR patients found that a preventable ADR was present in almost 20% of the cases, especially in patients aged ≥70 years (Schmiedl et al., 2018). However, only 3 of 71 patients admitted by an ADR suffered a medication error (4.2%) in our study.

The errors in the prescription or use of drugs, such as those we have detected in this study, could be solved with measures available to health personnel. These measures include resources and time investment for a deeper education of patients about their disease, the establishment of double control over prescriptions, as well as encouraging the use of a single prescription module (already in use in Community of Madrid, Spain). Currently there are no adequate tools to assess the risk of ADR in primary care. Further research is needed on tools to identify high-risk patients for the prevention of ADR-related hospital admissions (Parameswaran Nair et al., 2016).

When trying to avoid medication errors, it is vital to ensure that the correct medication is prescribed for the correct patient, in the correct dosage, *via* the correct route and timed correctly.

The Patient Safety Strategy of the Spanish National Health System made a series of recommendations to promote the safe use of medicines (Patient Safety Strategy of the National Health System, 2015-2020). These recommendations include, among others, (i) the implementation of electronic prescription programs, including clinical decision support systems which should be integrated into the information systems of the health center, and available to all professionals involved in the patient care; (ii) the standardization of preparation and administration procedures of injectable drugs and parenteral nutrition; (iii) the systematical review of the medication in chronic polymedicated patients, to detect or prevent adverse events, ensure adequacy, and improve adherence to treatment; (iv) the establishment of specific interventions aimed at avoiding errors in medication (training, dissemination of guidelines, etc.); (v) the development of training actions for healthcare professionals on the safe use of medicines, information and training programs for patients and caregivers; (vi) promote notification as a tool to disseminate the culture of patient safety.

### Study Limitations

During the development of this work a series of limitations have risen, such as the selection bias that might have occurred when limiting the study to one month, in a single hospital in Madrid and where, in addition, the population is slightly heterogeneous. As a consequence, the data obtained could only be extrapolated to a population with similar characteristics. Although the sample is relatively small (847 patients), it is superior to that of other published studies and enough to estimate the incidence accurately. We have not performed a stratified analysis by different groups because our objective was to assess how many hospital admissions are due to ADR, not how many patients in a certain group who are seen in the emergency department require admission. Moreover, in relation to the causality algorithm application, it is necessary to highlight that there is no internationally validated algorithm and that the algorithm of Spanish pharmacovigilance system is only one tool for evaluation. This type of algorithms depends closely on the clinical experience and the judgement of the evaluator.

## Conclusions

8.4% (95% CI 6.5%–10.3%) of emergency admissions were ADR-related, most of which were produced at the therapeutic dose, and this could be explained by the advanced age of our patients and the high prevalence of comorbidities and polymedication.

The most frequently reported ADR have been opportunistic infections resulting from antineoplastic and immunomodulatory treatments, and bleeding caused by antiaggregation and, to a lesser extent, anticoagulant treatments.

Serious ADR account for a significant percentage of emergency admissions and often affect a fragile and potentially fatal population group. Therefore, measures should be established for stricter control over these patients, monitoring their pathologies and reducing the number of drugs to the minimum possible.

We found 0.6% (0.08%–1.12%) of emergency admissions related with medication errors. Although they are difficult to detect, they are preventable. The solution is within the reach of health personnel, establishing measures such as: improving education about the disease for patients and the establishment of double control over prescriptions.

## Data Availability Statement

The datasets generated for this study are available on request to the corresponding author.

## Ethics Statement

The studies involving human participants were reviewed and approved by the Ethics Committee for Research on Medicines (CEIm) of Hospital Universitario de La Princesa. Written informed consent for participation was not required for this study in accordance with the national legislation and the institutional requirements.

## Author Contributions

MS-R, GM, and FA-S wrote the manuscript. GM, BG, and FA-S designed the research. BG, GM, and DO performed research. MS-R, BG, and GM analyzed the data. FA-S and DO contributed with analytical tools.

## Conflict of Interest

FA-S and DO have been consultants or investigators in clinical trials sponsored by the following pharmaceutical companies: Abbott, Alter, Chemo, Cinfa, FAES, Farmalíder, Ferrer, GlaxoSmithKline, Galenicum, Gilead, Italfarmaco, Janssen-Cilag, Kern, Normon, Novartis, Servier, Silverpharma, Teva, and Zambon.

The remaining authors declare that the research was conducted in the absence of any commercial or financial relationships that could be construed as a potential conflict of interest.
